# LncRNA modulates Dpp-mediated wing development to influence flight in *Aedes aegypti*

**DOI:** 10.1007/s00018-026-06325-8

**Published:** 2026-06-30

**Authors:** Jianping Cao, Lin Ling

**Affiliations:** School of Life Science and Technology, The Key Laboratory of Developmental Genes and Human Disease, Southeast University, Nanjing, 210096 China

**Keywords:** lncRNA, miR-281, Dpp, Wing bud, Apoptosis, Flight

## Abstract

The flight capability of *Aedes aegypti* underpins global spread of vector-borne diseases (e.g., dengue, Zika, chikungunya), making it a key control target. Decapentaplegic (Dpp), evolutionarily conserved in insect morphogenesis, has unclear spatiotemporal regulatory networks in *Ae. aegypti* wing bud/haltere development. Here, we identify a wing bud/haltere-specific long non-coding RNA (lncRNA), AAEL025449, forming a competing endogenous RNA (ceRNA) axis to regulate Dpp-mediated development. Spatially, AAEL025449 acts as a microRNA-281 (miR-281) sponge, relieving Dpp transcriptional repression in wing buds. Temporally, it tunes Dpp dynamics during critical apoptosis windows, enabling Dpp to modulate apoptotic timing via the canonical c-Jun N-terminal kinase (JNK) pathway. Disruption of this axis results in shorter wings, indirectly compromising flight capacity and reducing mating success, thereby linking molecular perturbations to fitness-related traits. Additionally, it further clarifies the key temporal-spatial nodes where Dpp regulates wing bud cell apoptosis via JNK, with the spatial node differing from conventional Dpp regulation of wing bud development and manifesting as a gradient diffusion from anterior to posterior wing bud margin during the critical apoptosis period in *Ae. aegypti*. Our study uncovers a novel spatiotemporal regulatory mechanism for wing bud plasticity, advancing insights into insect wing bud adaptive evolution. This ceRNA axis provides a foundation for selecting targets to suppress mosquito populations and mitigate disease transmission.

**Supplementary Information:**

The online version contains supplementary material available at https://doi.org/10.1007/s00018-026-06325-8.

## Introduction

The wings and homologous halteres of *Ae. aegypti* are critical for its flight-dependent survival, reproduction, and role as a disease vector [1–3]. Their morphology is primarily shaped by genetic determinants, rendering them critical for understanding mosquito ecology and developing targeted vector control strategies [4–6]. The development of wing buds and halteres in Diptera insects, during the pupal stage, involves a strictly hormone and genetically regulated apoptotic window that is crucial for the normal formation of adult wings [7–12].

Decapentaplegic (Dpp), a member of the TGF-β/BMP family, is a key morphogen that modulates the development of multiple tissues and organs (including wing buds) in insects [13–16]. In Diptera, halteres are modified hindwings, and Dpp regulates the size development of this series of homologous organs [1, 17, 18]. Dpp signaling is temporally regulated: promoting cell proliferation and growth in early development, specifying cell fate and differentiation in late pupae [15, 19, 20]. Dpp also acts as a survival factor, preventing apoptosis (programmed cell death) in wing cells [21, 22]. During development, cells compete for Dpp; those that fail to receive sufficient Dpp upregulate the *brinker* (*brk*) gene, activating JNK-dependent apoptosis and ensuring only viable cells contribute to the final wing structure [21, 22]. Thus, Dpp orchestrates a balance between growth, patterning, and cell survival—with precise spatial and temporal regulation being critical for normal wing development and the prevention of excessive apoptosis during the pupal stage [21, 22]. Nonetheless, the regulatory mechanism of Dpp in *Ae. aegypti* wing development has scarcely been explored, which represents a promising vector control target.

Long non-coding RNAs (lncRNAs) are RNA molecules longer than 200 nucleotides that do not encode proteins [23–25]. microRNAs (miRNAs) are small non-coding RNAs, typically approximately 22 nucleotides in length, that regulate gene expression by binding to complementary sequences on target messenger RNAs (mRNAs), leading to mRNA degradation or translational inhibition [23–25]. A key mechanism by which lncRNAs modulate gene expression is by acting as ceRNAs. In this role, lncRNAs function as “sponges” for miRNAs [26–30]. They competitively bind to miRNAs via shared miRNA response elements, sequestering miRNAs and preventing them from interacting with their target mRNAs. This process alters the availability of miRNAs to repress target transcripts, ultimately modulating the expression of those mRNAs [26–30]. The ceRNA network formed by lncRNA/miRNA/mRNA interactions is increasingly recognized as a critical regulatory layer in gene expression, in which expression pattern-specific non-coding RNAs (ncRNAs) are considered highly promising molecular targets [26–30]. However, current research on Dpp-related ceRNA regulatory mechanisms remains limited. Given the broad regulatory roles of Dpp in insect tissues, Dpp-targeted interventions lack sufficient specificity, thus necessitating exploration of specific upstream targets in *Ae. aegypti* by leveraging ceRNA regulatory potential. Here, we aim to identify potential components precisely regulating Dpp within the ceRNA network, to provide eco-friendly targets for vector control.

This study elucidates a spatiotemporal regulatory paradigm governing wing bud plasticity in *Ae. aegypti*, wherein the wing/haltere-specific lncRNA AAEL025449 (AAEL025449) operates as a ceRNA to fine-tune Dpp-mediated apoptotic timing during pupal development. By acting as a miR-281 sponge, AAEL025449 spatially preserves Dpp availability in wing buds while temporally modulating its dynamics through the JNK pathway, ensuring precise cell fate decisions during the critical apoptosis window. Functionally, disruption of this axis causes wing truncation, which in turn compromises flight dynamics (lift, maneuverability, and endurance) and diminishes mating competitiveness—collectively posing a triple threat to vectorial capacity. Collectively, these findings unveil a potential regulatory node for precision vector control.

## Materials and methods

### Mosquito rearing

Eggs were placed on humidified filter paper. Three days later, they were transferred to deionized water and incubated in a low-pressure incubator at 66 kPa for 30 min to achieve synchronized hatching. Larvae (with a developmental stage duration of approximately 7 days) and pupae (with a developmental stage duration of approximately 2 days) were reared in water at 27 °C and fed commercial fish food pellets (Yee); no feeding was provided during the pupal stage. Adult mosquitoes were maintained in cages with temperature controlled at 27 °C and relative humidity at 80%, and supplied with 10% sucrose solution and sterile water. Blood feeding for adult females was performed using White-feathered broilers. All procedures involving vertebrates adhered to the guidelines of the Experimental Ethics Committee of Southeast University.

### Quantitative real-time PCR

For wing bud and haltere samples, each contained 100 wing buds or and halteres; for other samples, each included 10 tissues or individuals. Total RNA was extracted from samples using TRIzol reagent (Vazyme), followed by DNase I treatment (NEB). RNA purity was assessed by measuring the A₂₆₀/A₂₈₀ ratio with a NanoDrop OneC Spectrophotometer (Thermo Fisher Scientific). A constant amount of total RNA (500 ng) was reverse-transcribed into cDNA using either the HiScript III RT SuperMix for quantitative real-time PCR (qPCR) (+ gDNA Wiper) kit or the miRNA First-Strand cDNA Synthesis SuperMix kit. Following 10-fold dilution, cDNA samples were amplified using Taq Pro Universal SYBR qPCR Master Mix (Vazyme) and analyzed on a CFX96 qPCR system (Bio-Rad) according to the manufacturer’s instructions. U6 or RPS7 was used as the internal reference gene, and relative expression levels were calculated via the 2^⁻ΔΔCt^ method. Primer sequences for qPCR are provided in Table S1. All qPCR experiments were performed with at least three biological replicates. Reagent specifications are summarized in Table S2.

### RNA FISH

Three-color FISH was employed to visualize the spatial localization of AAEL025449, miR-281, and Dpp in mosquito wing buds, following the protocol described by Guo et al. [31]. Briefly, wing bud tissue samples underwent sequential processing: fixation, dehydration, sectioning, pre-hybridization, hybridization, washing, counterstaining, and mounting. After completing all steps, samples were stored in the dark. Confocal laser scanning microscopy was performed using a Zeiss LSM 900 system (Carl Zeiss Microscopy GmbH). Fluorescence excitation was carried out at 555 nm (Cy3), 492 nm (FAM), and 650 nm (Cy5), respectively, and images were captured using ZEN 3.1 software (Zeiss).

Specific antisense RNA probes—labeled with Cy3 (5′-AAUCUGGUCUGAGGCAGCAC-3′) for AAEL025449, FAM (5′-FAM-ACUGUCGACGGAUAGCUCUC-3′) for miR-281, Cy5 (5′-AGAGUCCGUGGUUCUGCUUG-3′) for Dpp, Texas Red (5′-GCAACGAAGGAGGUCCUGAA-3′) for *brk*, Cy7 (5′-GCCGUGAAGAGUAGACCGAG-3′) for c-Jun—were used to detect each target. All probes were commercially synthesized by Sangon Biotech Co., Ltd. Scrambled probes (labeled with Cy3: 5′-Cy3-UUGUACUACUGUAAAGUACUG-3′; FAM: 5′-FAM-UUGUACUACUGUAAAGUACUG-3′); Cy5: 5′-FAM-UUGUACUACUGUAAAGUACUG-3′) for lncRNA, miRNA, and mRNA, respectively, served as negative controls.

### In vitro luciferase reporter gene assays

An ~ 70-bp fragment containing the predicted miR-281 target sites in AAEL025449 and Dpp was cloned into the psiCHECK-2 dual-luciferase reporter vector (Promega). Mutations were introduced to disrupt miRNA-binding specificity by mutating 20 nucleotides (nt) within the target sites—including the region complementary to the miR-281 seed sequence. Co-transfection assays in S2 cells were conducted following the protocol described by Yang et al. [32]. Transfected cells were analyzed per the Dual-Luciferase Reporter Assay System (Promega) kit instructions. Signal intensities were quantified at 480 nm (Renilla luciferase) and 560 nm (Firefly luciferase) using a Varioskan Flash Multimode Reader (Thermo Fisher Scientific). Detailed reagent information is listed in Table S2.

### Establishment of KO strains

The synthesis of single-guide RNAs (sgRNAs) was detailed in our previous study [33]. Primer sequences for sgRNA target design are provided in Table S1. Briefly, a mixture of sgRNAs (40 ng/µL) and Cas9 protein (333 ng/µL) was microinjected into pre-gastrulation embryos. Injected embryos hatched 5 days post-injection and were reared to adulthood using the previously described protocol [34]. Genotyping was performed by analyzing exuviae shed during the pupal stage of the first transgenic generation. Finally, two independent homozygous mutant strains (KO) were generated via efficient mutation screening and optimized germline transformation strategies [35]. Detailed reagent information is provided in Table S2.

### Injections during the pupal stage

The methodology for in vivo injection was described in detail elsewhere [36]. Mosquitoes were anesthetized on a cold plate and microinjected with either 1 µg/300 nL dsRNA targeting specific genes (for RNA interference) or 100 pmol of miRNA mimic/inhibitor (for miRNA overexpression or suppression) into the thoracic region. The injected mosquitoes were allowed to recover under standard rearing conditions (27 °C, 80% relative humidity) for 36 h. The efficiency of gene expression regulation was quantified via qPCR analysis. Primer sequences for the target genes are listed in Table S1. NC mimic, miR-281 mimic, NC inhibitor, and miR-281 inhibitor were synthesized by Sangon Biotech Co., Ltd.

### TUNEL staining

Apoptotic cell death rates were quantified using terminal deoxynucleotidyl transferase-mediated dUTP nick end labeling (TUNEL) assays. Wing bud tissues were processed following the manufacturer’s protocol for the APO One-Step TUNEL Apoptosis Kit (Ribo). Confocal microscopy was performed using a Zeiss LSM 900 system (Carl Zeiss Microscopy GmbH). Fluorescence excitation was carried out at 492 nm, and images were captured using ZEN 3.1 software (Zeiss).

### Flight index detection

This section detects horizontal flight speed, lift, and turning flight capability using an open glass tube apparatus. For horizontal flight speed detection, a 1 m long and 10 cm in diameter glass tube is horizontally connected to a rearing cage. ~72 h post-eclosion (PE) female mosquitoes are selected as subjects. A hand is placed at the other end of the tube to continuously apply an attractive stimulus. Timing starts when the first female enters the tube, and the number of females reaching within 10 cm of the hand at different time points is recorded for 30 s to measure horizontal flight speed. For lift detection, the apparatus is vertically positioned using a glass tube with a side branch. A horizontal breeze (0.5 m/s) is introduced from the side branch, ensuring a weaker longitudinal breeze. Lift is measured by applying the same attractive stimulus and standard timing (recording for 50 s). For turning flight detection, a glass tube with a 90° side branch (both main and side branches horizontal) is assembled. After continuously attracting with a hand for 30 s, the number of females distributed in different regions is recorded.

This section records free flight distribution and sustained flight duration using a closed glass tube apparatus. A 1 m long and 20 cm in diameter glass tube has its bottom sealed and a removable cardboard placed at the top. A mixed-sex *Ae. aegypti* container (20 cm diameter opening) is inverted over the cardboard. After the mosquito colony becomes active, the cardboard is removed, allowing the group to freely fly in the closed apparatus for 5 min, during which individual distribution zones are recorded. Random sampling is performed during this period to record sustained flight duration.

### Flight video recording

Mosquitoes (~ 72 h PE) from the same group were immobilized by cooling on ice for 10 min, then transferred to the bottom of the two arms of a Y-tube. They recovered in a temperature (27 °C)- and humidity-controlled incubator for 3 min. The Y-tube was positioned vertically with its arms downward and main tube upward. Once mosquitoes had fully recovered, a mosquito attractant lure was placed at the top of the main tube. Mosquito movement trajectories were recorded for > 10 s, and videos were played back at double speed.

### Mating success assessment

Male and female pupae were reared separately. Post-eclosion, each group comprised 10 females and 10 males, maintained in cages with access to sterile water and 10% sucrose solution until 72 h PE. A 30-min blood meal was then provided. At 72 h post-blood meal (PBM), each female was individually transferred to a ventilated petri dish lined with humidified filter paper, and egg-laying was induced under dark conditions for 2 h. Eggs were incubated for 72 h to achieve synchronized hatching. Mating success was defined as the proportion of egg-laying females that produced viable offspring. Ten biological replicates were performed for each experimental treatment.

### Lipid droplet staining

Fat body tissues were incubated in Nile red staining solution (20% glycerol in PBS containing a 1:10,000 dilution of 10% Nile red stock solution in dimethyl sulfoxide [DMSO]) for 15 min at room temperature. Confocal microscopy was performed using the Zeiss LSM 900 system (Carl Zeiss Microscopy GmbH). Excitation was carried out at 543 nm, and images were captured using ZEN 3.1 software (Zeiss). Detailed reagent information is provided in Table S2.

### Statistical Analysis

All data are presented as the mean ± standard error of the mean (SEM). Comparisons between groups were performed using the Student’s t test, with significance levels defined as follows: *p < 0.05, **p < 0.01, and ***p < 0.001. Statistical analyses were performed using GraphPad Prism version 8.

## Results

### Dpp regulates lipid degradation and body-size development during the pupal stage

To clarify the role of Dpp in the critical period of wing bud development in *Ae. aegypti*, we generated a Dpp interference model by injecting dsDpp (iDpp; targeting Dpp) or dsLuciferase (iLuc; negative control) at 0 h post-pupation (PP). qPCR analysis of temporal expression profiles in wing buds of both sexes confirmed successful model establishment: Dpp expression was significantly lower in iDpp compared to iLuc at 24 h PP (Supplementary Fig. S1A).

**Fig. 1:**
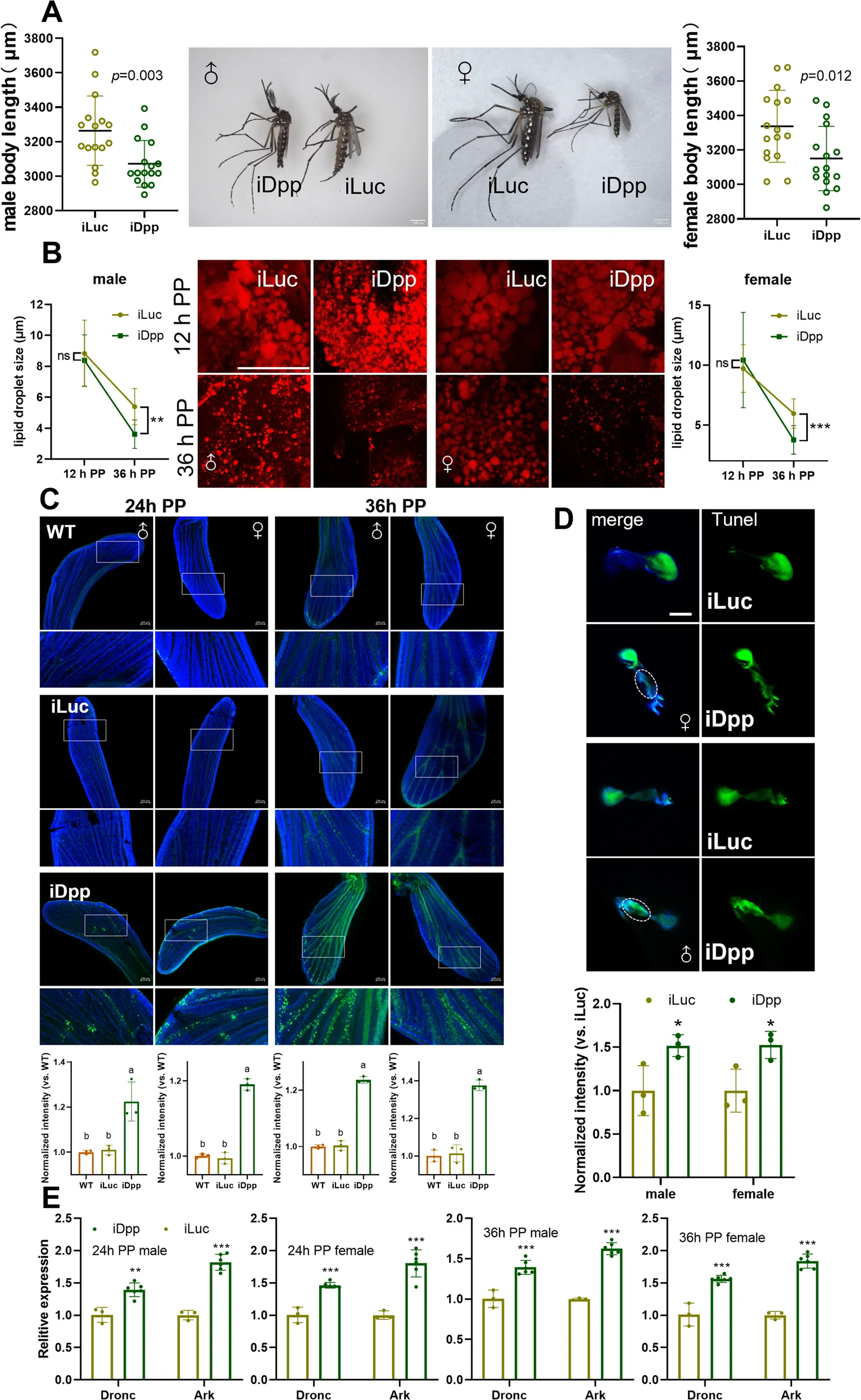
Interference with Dpp impairs pupal development in *Ae. Aegypti*. **A** Analysis of adult body size in iLuc and iDpp mosquitoes at 0 h PE (scale bar: 1000 μm; *n* = 16). **B** Confocal microscopy imaging visualizes lipid droplet accumulation in fat bodies of iLuc and iDpp at 12 h and 36 h PP (Scale bar: 100 μm). **C** Wing bud apoptosis was assessed using TUNEL-based apoptosis detection (scale bar: 100 μm; blue fluorescence: DAPI; green fluorescence: TUNEL). **D** Haltere apoptosis was assessed using TUNEL-based apoptosis detection (scale bar: 100 μm; blue fluorescence: DAPI; green fluorescence: TUNEL). **E** Quantification of core programmed cell death regulator expression profiles in wing buds at different time points was performed via qPCR. Data are represented as means ± SEM. ‘ns’ indicates *p* > 0.05; ‘*’ indicates *p* < 0.05; ‘**’ indicates *p* < 0.01; ‘***’ indicates *p* < 0.001

Phenotypic analysis revealed that Dpp interference induced significant changes in body size in both sexes. Compared with iLuc controls, iDpp individuals exhibited significantly shorter body length, whereas the wing-to-body length ratio remained unchanged (Fig. 1A, Supplementary Fig. S1B). This size divergence emerged by 36 h PP during pupal development (Supplementary Fig. S1C). As Dpp deficiency accelerates lipid degradation in the fat body, which in turn impacts body-size development during the pupal-to-adult transition in insects [37], we stained lipid droplets in the fat body at 12 h and 36 h PP to quantify lipid degradation rates (Fig. 1B). The results demonstrated that iDpp exhibited accelerated lipid degradation relative to iLuc controls. This finding explains our observations of body-size changes.

Since Dpp is required for precise apoptosis regulation during wing development [21, 22], we employed TUNEL staining to label apoptotic cells in wing buds across developmental stages. Results showed that iDpp had significantly higher wing bud cell apoptosis than wild-type (WT) and iLuc at 24 and 36 h PP, similar patterns were observed in haltere stalk cells at 36 h PP (Fig. 1C and D). Validation with apoptosis marker genes further supported this: iDpp displayed significantly increased expression of the cell death activators Dronc and Ark at 24 h and 36 h PP (Fig. 1E). These indicate Dpp deficiency accelerates apoptosis during early wing bud and haltere development.

### LncRNA AAEL025449 functions as a spatiotemporally defined ceRNA to regulate the miR-281/Dpp axis

**Fig. 2:**
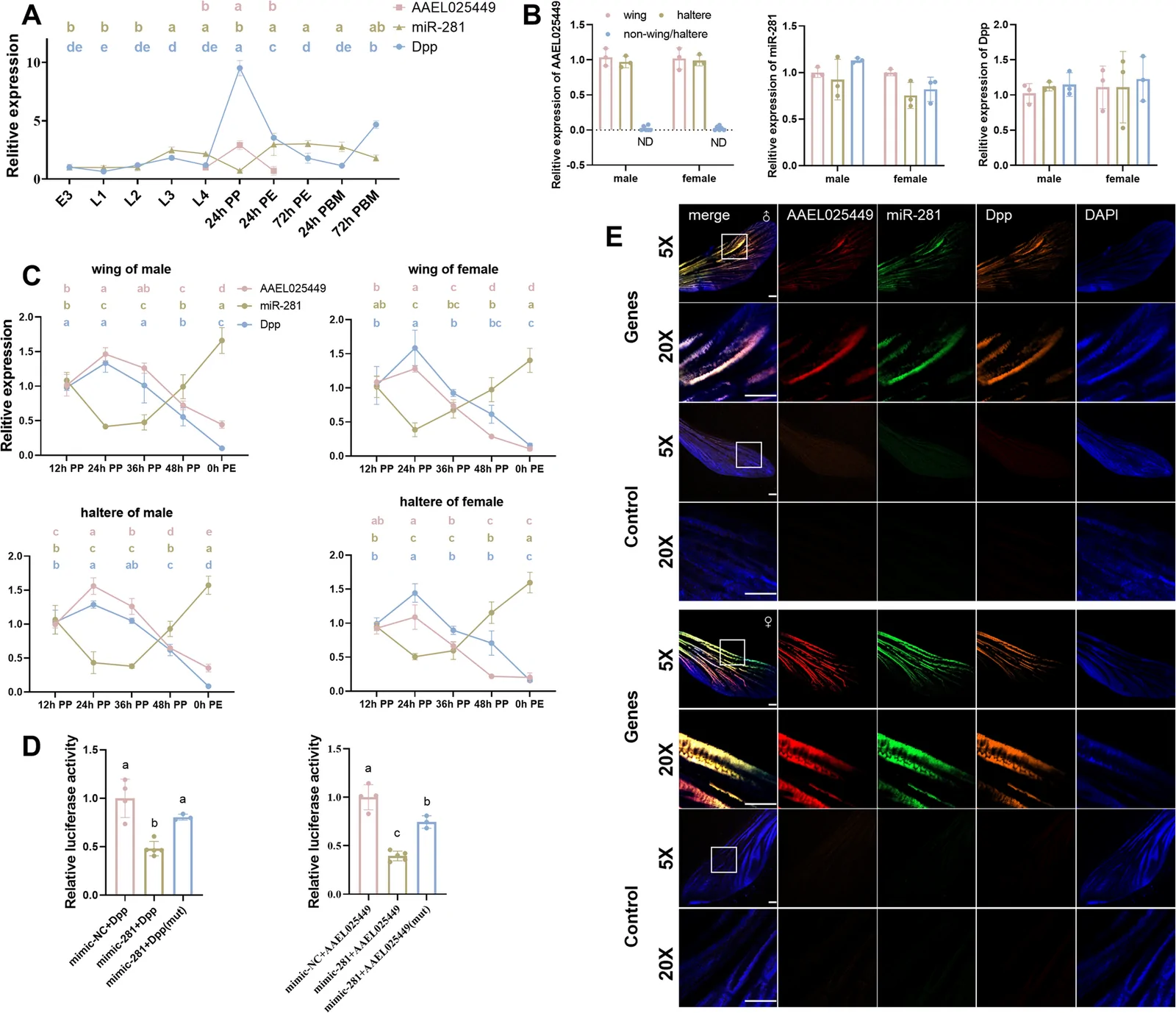
Competitive binding of AAEL025449 and Dpp to miR-281 in wings. **A** Temporal expression profiles of AAEL025449, miR-281, and Dpp across developmental stages (eggs [E], larvae [LV]) and time points (24 h post-pupation [PP], 24/72 h post-eclosion [PE], 24/72 h post-blood meal [PBM]). **B** Comparison of AAEL025449, miR-281, and Dpp expression in wing bud/haltere versus non-wing bud/haltere tissues at 24 h PP. **C** Dynamic expression changes of AAEL025449, miR-281, and Dpp in wing buds and halteres during different pupal stages. **D** The dual-luciferase reporter assay demonstrates in vitro miR-281-targeted binding to AAEL025449 and Dpp. **E** Fluorescence in situ hybridization (FISH) reveals colocalization of AAEL025449, miR-281, and Dpp in vivo (scale bar: 100 μm). Data are represented as means ± SEM. ‘ND’ indicates Not Detected

From the miRNA and lncRNA repertoire of *Ae. aegypti*, we constructed a putative ceRNA network centered on Dpp via miRanda (http://www.microrna.org/microrna/home.do) and RNAhybrid (https://bibiserv.cebitec.uni-bielefeld.de/rnahybrid)-mediated predictions and minimum free energy (mfe)-based screening. To identify tissue-specific regulators, we performed quantitative expression profiling of the top-five ranked miRNA-lncRNA pairs (predicted by RNAhybrid) in wing bud versus non-wing bud tissues at 24 h PP. Results showed that AAEL025449 was markedly under expressed in non-wing bud tissues compared to wing buds, with undetectable levels in some replicates (Supplementary Fig. S2). In contrast, other tested ncRNAs exhibited no obvious tissue specificity (Supplementary Fig. S2). Combined with target prediction data, we inferred that AAEL025449 may competitively sequester miR-281, thereby alleviating the Dpp 3’ untranslated region (UTR) repression mediated by potential wobble pairing (Supplementary Fig. S3) [38, 39]. First, we quantified the expression levels of these three genes in intact *Ae. aegypti* individuals (female: male = 1:1) across different developmental time points using qPCR. Results showed stage-specific expression of AAEL025449 exclusively at the fourth larval (L4), 24 h PP, and PE stages, while miR-281 and Dpp were ubiquitously expressed throughout all developmental stages. Notably, AAEL025449 and Dpp were significantly upregulated at 24 h PP relative to other stages, whereas miR-281 expression was significantly reduced at this stage compared to adjacent developmental stages (Fig. 2A). Collectively, these data revealed a negative correlation between AAEL025449/Dpp and miR-281 expression, with all three genes exhibiting prominent expression changes at 24 h PP.

Next, we measured the expression of these three genes in the wing buds and halteres versus non-wing bud/haltere tissues of male and female pupae at 24 h PP. The results showed AAEL025449 was specifically expressed in wing buds and halteres of both sexes, with comparable levels between the two. In contrast, miR-281 and Dpp were detected in both wing bud/haltere and non-wing bud/haltere tissues, showing no significant differences across tissues or sexes (Fig. 2B). To directly visualize correlations between these three genes and wing bud/haltere development, we profiled their expression across developmental stages. Results showed that, irrespective of sex, AAEL025449 and Dpp were highly expressed from 12 to 36 h PP in wing buds, peaking at 24 h PP, while miR-281 displayed the inverse pattern (Fig. 2C). Combined with Fig. 2B, given their high similarity in both expression levels and trends between haltere and wing bud across different developmental time points during the pupal stage, we will prioritize wing buds as the primary detection target in subsequent studies. Key findings from halteres will be used to strengthen the evidence, supporting our conclusions. Combined, these findings indicate that AAEL025449 is a spatiotemporally specific lncRNA associated with *Ae. aegypti* wing bud and haltere development. Its expression is negatively correlated with miR-281 expression and positively correlated with Dpp expression, and these correlated expression patterns conform to the regulatory principles of ceRNAs [26, 29]. Based on this, we examined the effects of Dpp interference on AAEL025449 and miR-281. Results showed that AAEL025449 expression was significantly reduced and miR-281 expression was significantly increased in iDpp wing buds at 24 h PP versus iLuc (Supplementary Fig. S4), supporting a model wherein reduced Dpp elevates free miR-281 levels to enhance its binding-mediated suppression of AAEL025449, thereby establishing a ceRNA regulatory axis among these three genes.

To further confirm the presence of regulatory interactions among these three genes, we generated four reporter plasmids targeting the potential binding sites, including wild-type (AAEL025449/Dpp) and mutant (mut) target fragments of AAEL025449/Dpp, respectively, and cloned them into the XhoI site of the psiCHECK2 vector (Supplementary Fig. S5). Luciferase activity assays conducted in *Drosophila* Schneider 2 (S2) cells demonstrated that co-transfection with miR-281 mimics significantly reduced the expression of reporter genes containing AAEL025449 or Dpp targets relative to the mimic-NC negative control, whereas mutagenesis of the miR-281 seed sequence targets in the reporter restored the luciferase activities of both (Fig. 2D), confirming sequence-specific target recognition. To further directly verify this interaction in wing bud tissue, we performed fluorescence in situ hybridization (FISH). Results demonstrated that AAEL025449, Dpp, and miR-281 transcripts co-localized in wing bud tissue and were predominantly expressed in the dorsal wing bud veins (Fig. 2E), providing direct evidence for their spatial colocalization and functional interaction. Combined, the spatiotemporal expression profiles, target validation, and spatial localization data establish that AAEL025449 functions as a spatiotemporally specific ceRNA regulating the miR-281/Dpp axis.

### AAEL025449 preserves apoptotic timing during pupal wing bud development

**Fig. 3:**
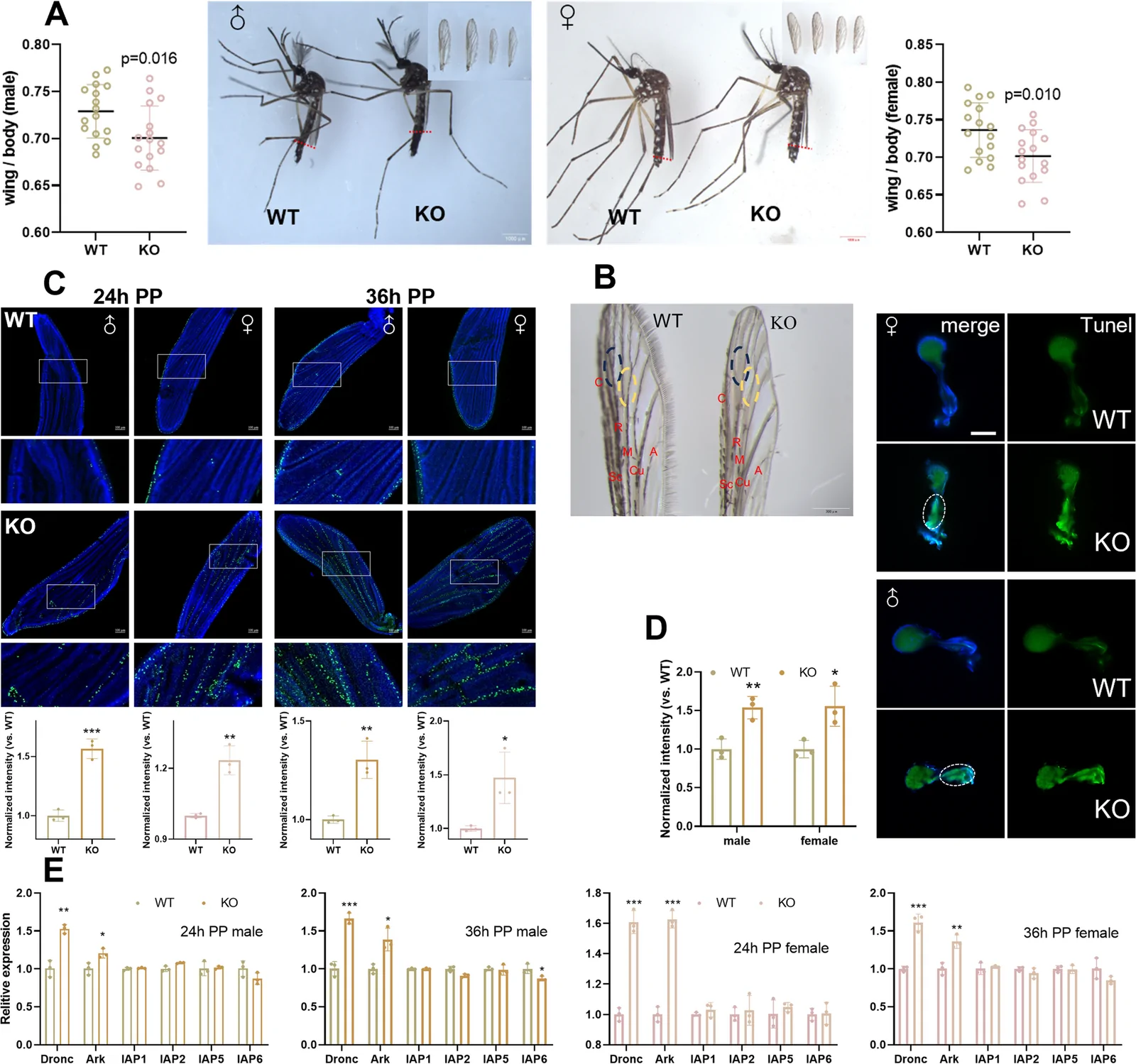
CRISPR-Cas9-mediated disruption of the AAEL025449 locus impairs pupal wing development. **A** Histological imaging of adult bodies and wings, and statistical analysis of wing-to-body length ratios, in wild-type (WT) and knockout (KO) mosquitoes at 0 h PE (scale bar: 1000 μm; *n* = 16). **B** Comparison of wing vein distribution between WT and KO (costa[C], subcostal [Sc], radius [R], media [M], cubitus [Cu], anal veins [A]). **C** Wing bud apoptosis was assessed using TUNEL-based apoptosis detection (scale bar: 100 μm; blue fluorescence: DAPI; green fluorescence: TUNEL). **D** Haltere apoptosis was assessed using TUNEL-based apoptosis detection (scale bar: 100 μm; blue fluorescence: DAPI; green fluorescence: TUNEL). **E** Quantification of core apoptotic regulator expression profiles in wing buds at different time points was performed via qPCR. Data are represented as means ± SEM. ‘ns’ indicates *p* > 0.05; ‘*’indicates *p* < 0.05; ‘**’ indicates *p* < 0.01; ‘***’ indicates *p* < 0.001

To evaluate the impact of AAEL025449 on wing bud development, we generated *Ae. aegypti* AAEL025449 mutants using the CRISPR/Cas9 system. We injected embryos with a mixture of sgRNAs designed to target the miR-281 binding site and recombinant Cas9 protein at 2 h post-oviposition. Approximately 29% of injected embryos survived to adulthood, with a male-to-female ratio of 79:73. Sanger sequencing identified two distinct deletion strains (KO) in the progeny: -5 bp and − 7 + 4 bp deletions (Supplementary Fig. S6A). qPCR confirmed the successful generation of AAEL025449-deficient strains, although residual transcription persisted at low levels, as has been observed in other studies (Supplementary Fig. S6B) [40–44]. Subsequent experiments primarily focused on the miR-281 target deletion strain (-5 bp) as the core experimental model. Systematic phenotypic analysis revealed that KO individuals had body sizes comparable to WT counterparts but exhibited significantly smaller wing buds and a markedly disrupted wing-to-body (measured from the anterior end of the thorax to the posterior end of the abdomen) length ratio (Fig. 3A, Supplementary Fig. S7A, S7B). Closer inspection showed that ~ 20% of KO individuals displayed wing vein defects relative to WT, including reduced branching of accessory veins in the radius (R) and media (M), smaller wing cell size, and decreased wing trichome density (Fig. 3B).

To investigate the underlying causes of these phenotypes, we initially assessed miR-281 and Dpp expression in KO at 24 h PP. Results demonstrated significantly higher miR-281 expression and lower Dpp expression in KO individuals versus WT (Supplementary Fig. S8), suggesting AAEL025449 deficiency induces these phenotypes via dysregulation of the miR-281/Dpp axis. Results from TUNEL staining of wing buds demonstrated that apoptotic cells accumulated gradually, expanding from the wing margin to veins and finally filling wing bud cells. Compared to WT, both male and female KO showed significantly more apoptotic cells at 24 h and 36 h PP, with earlier and more abundant apoptotic cells in intermediate veins and wing bud cells (Fig. 3C). However, no significant differences in apoptosis were observed between WT and KO at 48 h PP or 0 h PE (Supplementary Fig. S9). In halteres at 36 h PP, regardless of sex, apoptosis in KO stalk cells was significantly higher than in WT individuals (Fig. 3D). These indicate AAEL025449 deficiency accelerates apoptosis during early wing bud and haltere development. In KO wing buds, accelerated apoptosis was accompanied by significantly increased expression of the core apoptosis activators Dronc and Ark, with no changes at 48 h PP or 0 h PE. In contrast, most apoptosis inhibitors (IAPs) showed no consistent significant changes across stages (Fig. 3E, Supplementary Fig. S10). Collectively, these data demonstrate that AAEL025449 deficiency reduces *Ae. aegypti* wing size by accelerating apoptosis at 24 h and 36 h PP.

### miR-281 mediates AAEL025449-derived regulatory signals

**Fig. 4:**
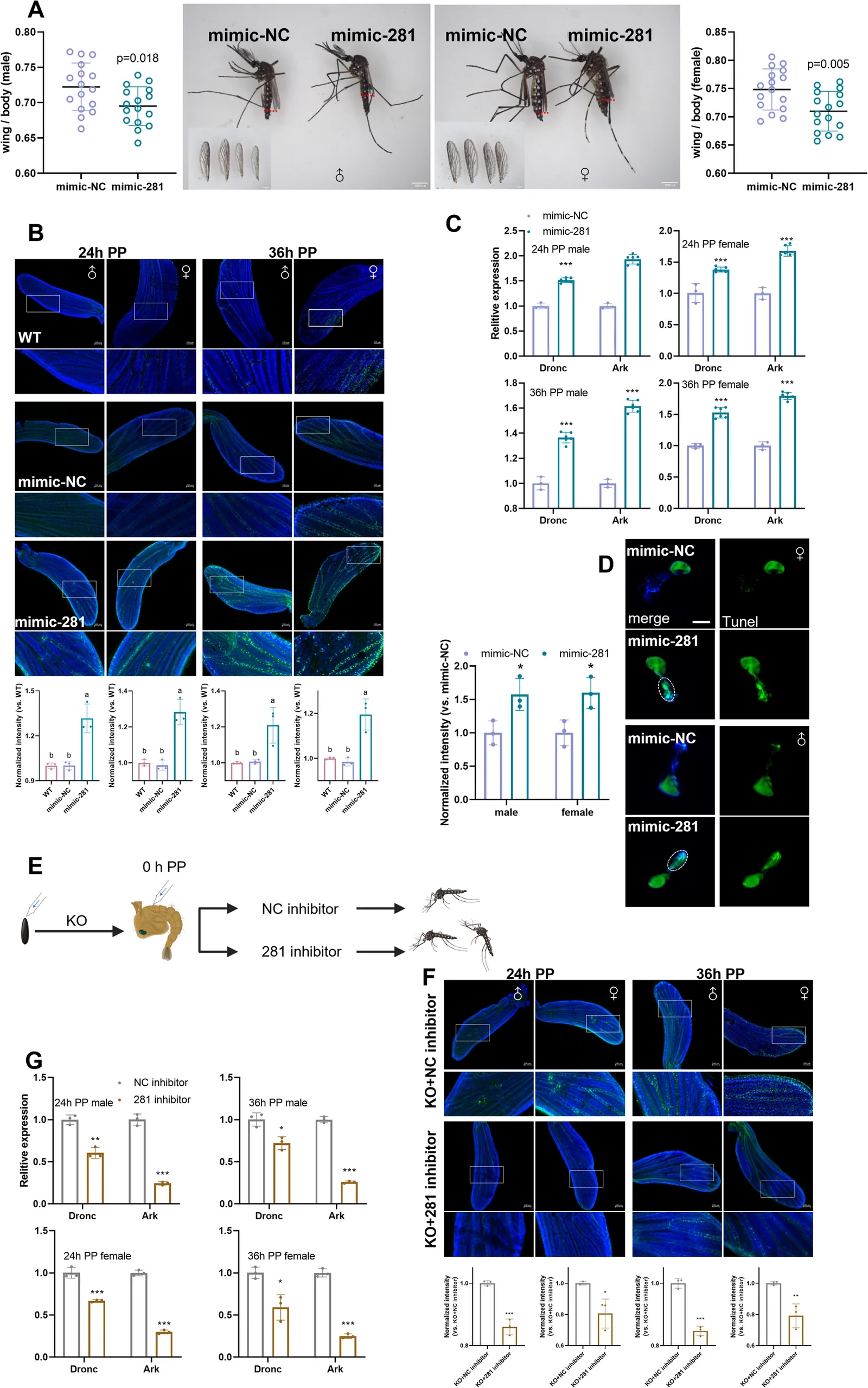
Overexpression of miR-281 recapitulates the effects of AAEL025449 deficiency on wing development. **A** Histological imaging of adult bodies and wings, and statistical analysis of wing-to-body length ratios, in mimic-negative control (mimic-NC) and miR-281 mimic (mimic-281) mosquitoes at 0 h PE (scale bar: 1000 μm; *n* = 16). **B** Wing bud apoptosis was assessed using TUNEL-based apoptosis detection (scale bar: 100 μm; blue fluorescence: DAPI; green fluorescence: TUNEL). **C** Quantification of core programmed cell death regulator expression profiles in wing buds at different time points was performed via qPCR. **D** Haltere apoptosis was assessed using TUNEL-based apoptosis detection (scale bar: 100 μm; blue fluorescence: DAPI; green fluorescence: TUNEL). **E** Schematic diagram of the rescue experiment. **F** Assessment of experimental intervention effects on wing bud apoptosis was performed using TUNEL-based apoptosis detection (scale bar: 100 μm; blue fluorescence: DAPI; green fluorescence: TUNEL). **G** qPCR quantification of core programmed cell death regulator expression profiles in wing buds at different time points. Data are represented as means ± SEM. ‘ns’ indicates *p* > 0.05; ‘*’ indicates *p* < 0.05; ‘**’ indicates *p* < 0.01; ‘***’ indicates *p* < 0.001

In the wing buds of KO individuals, miR-281 expression was significantly upregulated at 24 h PP (Supplementary Fig. S8). To determine if miR-281 acts as a downstream effector of AAEL025449, we overexpressed miR-281 in pupal *Ae. aegypti*. qPCR analysis of wing bud samples across developmental stages post-treatment confirmed successful establishment of our overexpression model: individuals injected with the negative control (mimic-NC, 100 pmol) at 0 h PP exhibited miR-281 wing bud temporal expression profiles similar to WT, whereas those injected with miR-281 mimic (mimic-281, 100 pmol) showed significantly higher miR-281 expression between 12 and 36 h PP compared to the mimic-NC group (Fig. 2C, Supplementary Fig. S11A). Consistent with the targeting relationship in this study, overexpressed miR-281 significantly suppressed AAEL025449 and Dpp expression in wing buds at 24 h PP (Supplementary Fig. S11B). Overexpression of miR-281 produced phenotypes similar to AAEL025449 deficiency: compared to the mimic-NC group, mimic-281-treated individuals had significantly shorter wings in both sexes despite comparable body sizes (Fig. 4A, Supplementary Fig. S11C). To elucidate the molecular basis underlying the distinct phenotypes observed in the iDpp compared with both KO and mimic-281, we compared Dpp expression in wing buds versus non-wing bud/haltere tissues across previous experimental groups. We found that AAEL025449 deficiency or miR-281 overexpression significantly suppressed Dpp expression in wing buds at 24 h PP (Supplementary Fig. S8, S11B). However, in non-wing bud/haltere tissues at 24 h PP, neither AAEL025449 deficiency nor miR-281 overexpression affected Dpp expression (Supplementary Fig. S12). When miRNAs share closely adjacent binding sites on a target mRNA, they competitively regulate the same gene, weakening individual regulatory capacity [45–47]. Based on this, we identified four miRNAs (miR-92b, miR-263a, miR-11895, and miR-989), among which miR-92b, miR-11,895, and miR-989 were significantly suppressed in the miR-281 overexpression model (Supplementary Fig. S13A). Wing-specific analysis showed all three were markedly downregulated in wings compared to non-wing tissues, with miR-92b and miR-989 nearly absent in wings (Supplementary Fig. S13B). This suggests these two miRNAs may alleviate miR-281-mediated repression of Dpp in non-wing tissues. Conversely, the diminished competition from these miRNAs in wings amplifies miR-281’s repression of Dpp. These results suggest that AAEL025449/miR-281 may act as wing bud/haltere-specific regulators of Dpp in *Ae. aegypti*.

Compared to mimic-NC, mimic-281 accelerated apoptosis in wing buds of both sexes between 24 and 36 h PP, primarily by inducing cell death mediated by the core apoptosis activators Dronc and Ark (Fig. 4B and C). mimic-281-treated haltere stalk cells exhibited significantly increased apoptosis at 36 h PP compared to mimic-NC (Fig. 4D). The temporal pattern of wing bud and haltere cell apoptosis induced by miR-281 overexpression closely resembled that induced by AAEL025449 deficiency, as described earlier.

To further confirm the specificity of miR-281 as a potential downstream effector of AAEL025449, we performed rescue experiments in KO: at 0 h PP, we injected KO individuals with either the negative control (KO + NC inhibitor, 100 pmol) or miR-281 inhibitor (KO + 281 inhibitor, 100 pmol) (Fig. 4E). Data with wing bud cell apoptosis as the core readout showed that compared to the KO + NC inhibitor group, the KO + 281 inhibitor group had significantly fewer apoptotic cells and lower Dronc/Ark expression at 24 and 36 h PP (Fig. 4F and G). These results indicate that inhibiting miR-281 in KO alleviated the accelerated wing bud cell apoptosis at 24 and 36 h PP. Together, these findings demonstrate that miR-281 acts as a specific downstream effector of AAEL025449 during pupal wing bud development in *Ae. aegypti*.

### AAEL025449/miR-281/Dpp sustains normal apoptotic timing via the JNK pathway

**Fig. 5:**
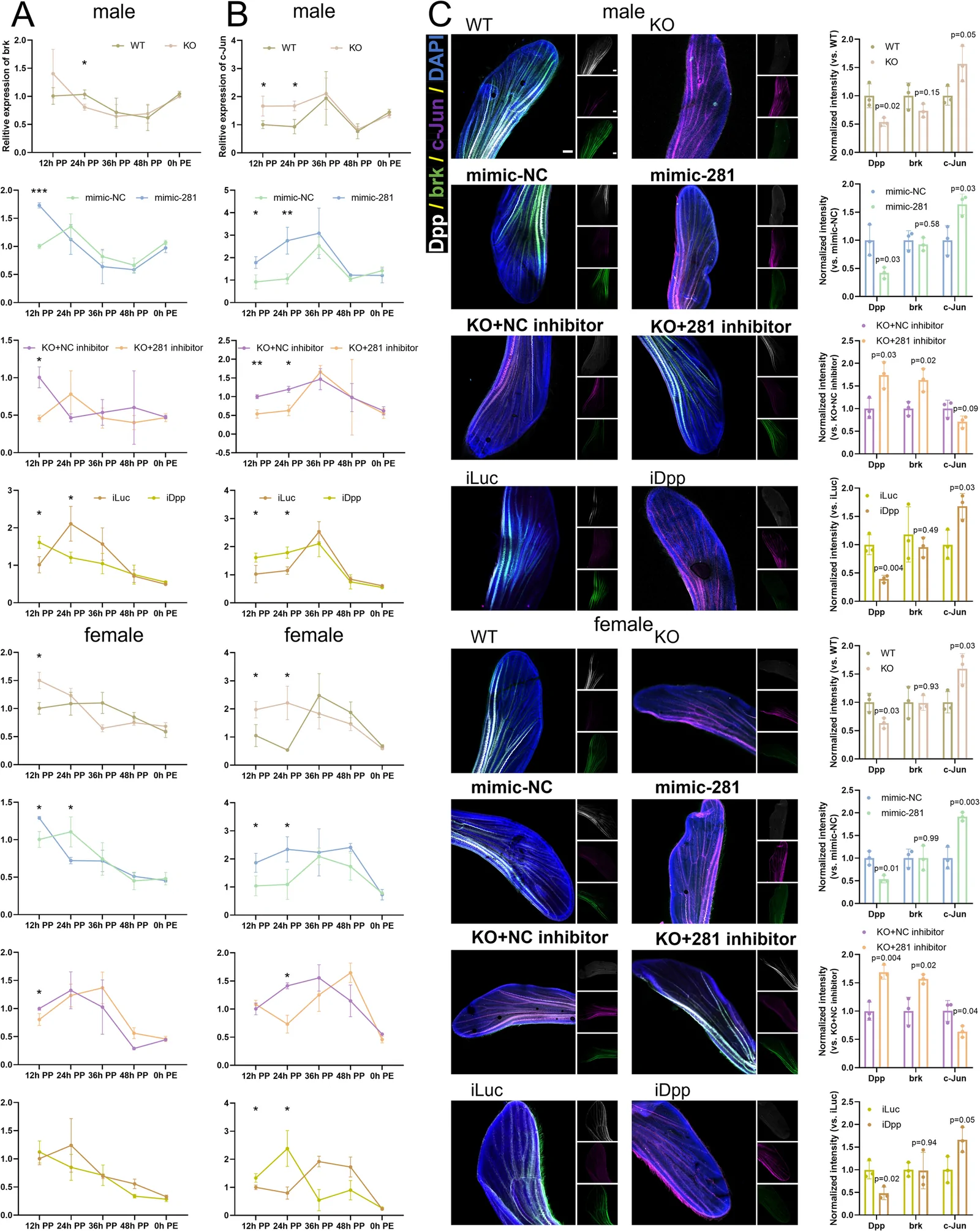
Temporal expression profiling of *brk* and c-Jun reveals the potential mechanism by which AAEL025449/miR-281/Dpp regulates wing development. **A–B** qPCR analysis of the effects of experimental intervention on *brk* and c-Jun expression. **C** FISH reveals pre-apoptotic cells and wing bud regions containing cells that have completed the JNK pathway (scale bar: 100 μm; blue fluorescence: DAPI; white fluorescence: Dpp; purple fluorescence: c-Jun; green fluorescence: brk). Significance analyses in Fig. 5 were performed by comparing experimental groups with controls at the same time point. Data are represented as means ± SEM. ‘ns’ indicates *p* > 0.05; ‘*’ indicates *p* < 0.05; ‘**’ indicates *p* < 0.01; ‘***’ indicates *p* < 0.001

Dpp, the downstream effector of our regulatory axis, suppresses the *brk* gene. When Dpp levels are low, *brk* is upregulated, activating c-Jun N-terminal kinase (JNK) and triggering apoptosis in cells with poor adaptability [21, 22, 48]. c-Jun expression contributed, to some extent, to the observed JNK-driven apoptotic phenotype [49–53]. Analysis of the temporal expression profiles of *brk* and c-Jun during wing bud development in each negative control group (WT/mimic-NC/iLuc) from this study showed that *brk* expression peaked in most samples at 24 h PP, whereas c-Jun expression reached its maximum at 36 h PP (Fig. 5A and B). This led us to hypothesize that the period of rapid wing bud cell apoptosis begins after 24 h PP, with a time delay in the regulation of downstream c-Jun by upstream *brk*, a delay common in other gene regulatory relationships [54–57].

Compared to controls, experimental groups (KO/mimic-281/iDpp) showed significantly upregulated *brk* at 12 h PP, followed by a gradual decrease. c-Jun in experimental groups maintained elevated expression between 12 h PP and 24 h PP (Fig. 5A and B). These results indicate that the experimental treatments accelerated apoptosis. Rescue experiments further strengthened our hypothesis: the *brk* and c-Jun expression trends in the KO + NC inhibitor control group were similar to those in the KO. Compared to KO + NC inhibitor, KO + 281 inhibitor restored *brk* and c-Jun expression patterns to near-WT levels (Fig. 5A and B). Between 36 h PP and 0 h PE, *brk* and c-Jun expression showed no significant differences across all experimental and control groups (Fig. 5A and B). This indicates that AAEL025449/miR-281/Dpp maintains normal apoptotic timing during the early to middle stages of pupal wing bud development in this study. FISH experiments further confirm the position of wing bud apoptosis at the critical time point of 24 h PP. The results show that in the control group (WT/mimic-NC/iLuc), pre apoptotic cells are primarily concentrated near the wing bud base at the anterior margin, gradually spreading from the anterior to posterior margin with a decreasing gradient. Meanwhile, in the experimental group (KO/mimic-281/iDpp), a large number of apoptotic cells that have completed the JNK pathway are already present at the anterior wing bud margin. These apoptotic cells are mainly localized on the subcosta (Sc), radius (R), and cubitus (Cu) veins (Fig. 5C). Thus, 24 h PP is identified as the critical turning point of the regulatory network proposed in this study.

### Flight stability and mating rate in *Ae. aegypti* are shaped by the AAEL025449/miR-281/Dpp-mediated wing development

**Fig. 6:**
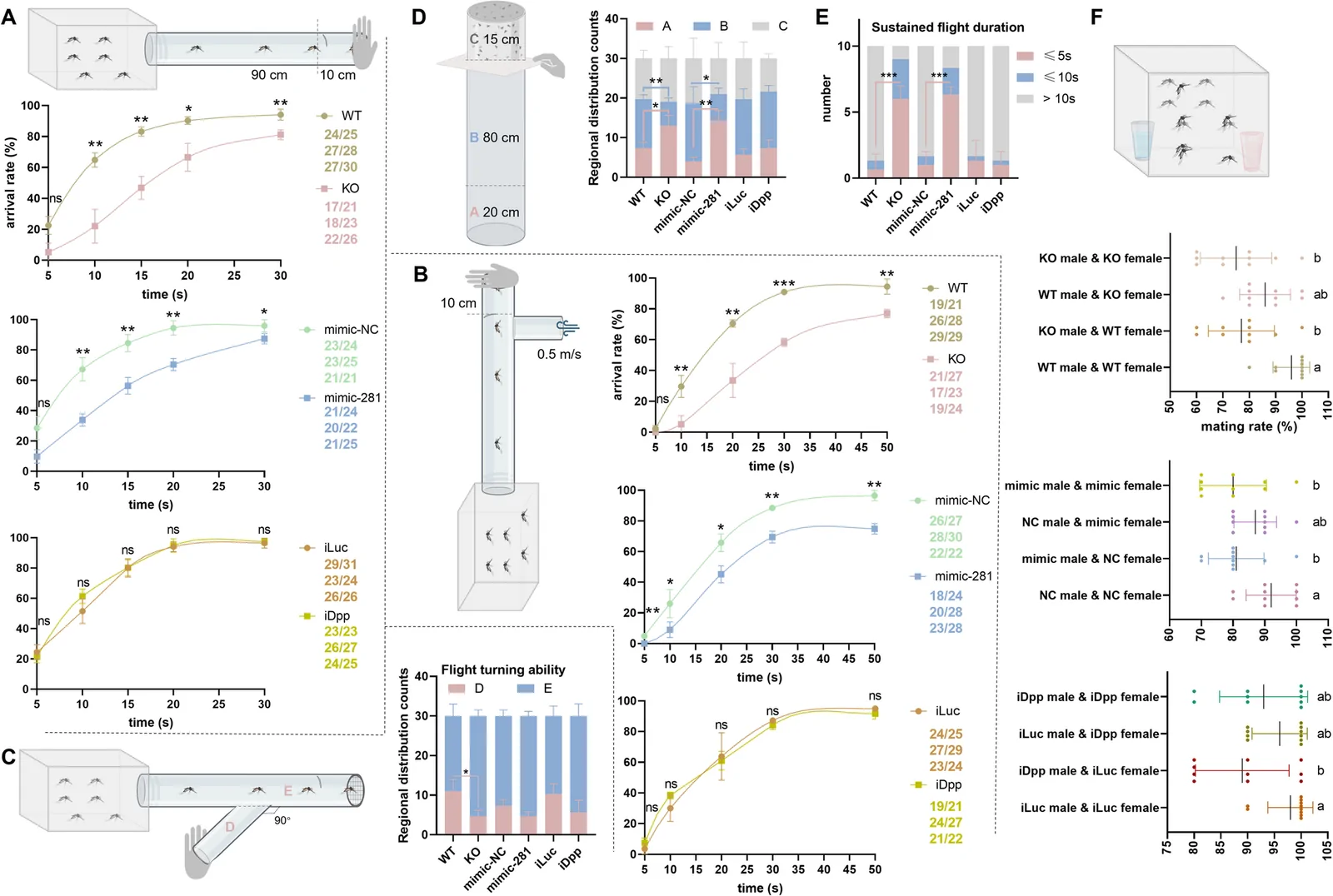
Behavioral assays of flight and reproductive capabilities. **A** Horizontal Flight Speed Assay: Recording the number of females reaching the blood source horizontally under windless conditions at different time points to characterize horizontal flight speed; significance analyses were performed by comparing experimental groups with controls at the same time point. **B** Flight Lift Assay: Recording the number of females reaching the blood source vertically under breezy conditions at different time points to characterize flight lift; significance analyses were performed by comparing experimental groups with controls at the same time point. **C** Flight Turning Ability Assay: Recording the distribution areas of 30 females after 30 s of flight to characterize flight turning ability. **D** Flight Altitude Assay: Recording the distribution areas of 30 individuals (female: male = 15:15) after 5 min of flight to characterize flight altitude. **E** Sustained Flight Duration Assay: Within the Fig. 6D experimental system, the sustained flight duration of 10 individuals (female: male = 5:5) was randomly recorded during a 5 min free flight period. **F** Mating Success Rate Assay: Mating success rates were analyzed for intragroup mating and intergroup cross-mating (female: male = 10:10) in controls and experimental groups (*n* = 10). Data are represented as means ± SEM. ‘ns’ indicates *p* > 0.05; ‘*’ indicates *p* < 0.05; ‘**’ indicates *p* < 0.01; ‘***’ indicates *p* < 0.001

In *Ae. aegypti*, individuals with smaller wings exhibit clear flight disadvantages compared to similar-sized counterparts in terms of lift, maneuverability, wingbeat frequency and power, and aerodynamic efficiency [58–61]. To clarify the implications of smaller wings for *Ae. aegypti* control, we performed behavioral assays. We evaluated the effects of experimental treatments on flight capability from five perspectives—female host-seeking flight time, lift, maneuverability, flight altitude, and sustained flight duration—and detailed our specific protocols in schematic diagrams and methodology sections. In host-seeking flight assays, under windless horizontal conditions (equal durations), AAEL025449-deficient and miR-281-overexpressing females showed significantly lower probabilities of reaching the host at all time points after 10 s and a significantly reduced proportion of individuals ultimately arriving (accounting for a smaller share of the total group) compared to controls (Fig. 6A). Under vertical flight with crosswind (0.5 m/s, equal durations), these groups also exhibited significantly lower arrival probabilities at most time points and a significantly reduced final arrival proportion (Fig. 6B). During windless 90° horizontal turning flight (30 individuals per group, 30 s free flight), KO females exhibited significantly fewer Zone D arrivals (host location) than WT, while mimic-281-treated females exhibited a reduction in Zone D arrivals compared to mimic-NC, though not statistically significant (Fig. 6C). These results indicate that AAEL025449/miR-281 indirectly affect female host-seeking flight speed, lift, and turning ability. In mixed-sex assays of flight altitude and sustained flight duration (15 males and 15 females, 5 min free flight), AAEL025449-deficient and miR-281-overexpressing groups had significantly more individuals in the bottom zone (Zone A) and fewer in the middle zone (Zone B) than controls, with some adhering to the inner wall of the rearing container (Zone C) without active flight (Fig. 6D). Concurrently, over 5 min, we randomly recorded sustained flight duration three times (10 individuals per recording), revealing significantly more individuals with sustained flight < 5 s in these groups versus controls (Fig. 6E). These findings reveal that AAEL025449/miR-281 indirectly affect *Ae. aegypti* flight altitude and sustained flight duration. Finally, we used synthetic human odor to induce flight and recorded videos to strengthen our conclusions. Among mosquitoes attracted to the odor source, fewer KO individuals reached the top of the Y-tube maze, and those that did took longer—specifically, some KO mosquitoes had limited flight elevation capacity, with insufficient height and duration (ascending briefly before falling downward) (Video S1, S2). Consistent with the KO, miR-281-overexpressing *Ae. aegypti* also showed reduced flight capability (Video S3, S4). Across all assay metrics, iDpp consistently showed no significant differences from iLuc. In summary, the wing-specific reduction caused by the AAEL025449/miR-281 axis compromises flight capacity in *Ae. aegypti*: with comparable body sizes, smaller wings reduce flight performance, thereby compromising female host-seeking ability.

Given that flight capacity is critical for successful mating in *Ae. aegypti*, we assessed mating rates as described above. Statistical analysis revealed that crosses between KO males and WT females had a significantly reduced mating rate, whereas crosses between WT males and KO females did not differ from WT controls. Overall, compared to WT male-female matings, the presence of KO males in mating pairs resulted in a significantly lower mating rate. Additionally, miR-281-overexpressing groups exhibited a similar pattern of change in mating rate relative to controls. In Dpp knockdown treatments, the only significant difference observed was a reduced mating rate in iDpp male & iLuc female crosses compared to iLuc male & iLuc female crosses (Fig. 6F). These findings suggest that, in this study, the disadvantage of reduced male flight capability or smaller body size directly impacts mating success.

## Discussion

**Fig. 7:**
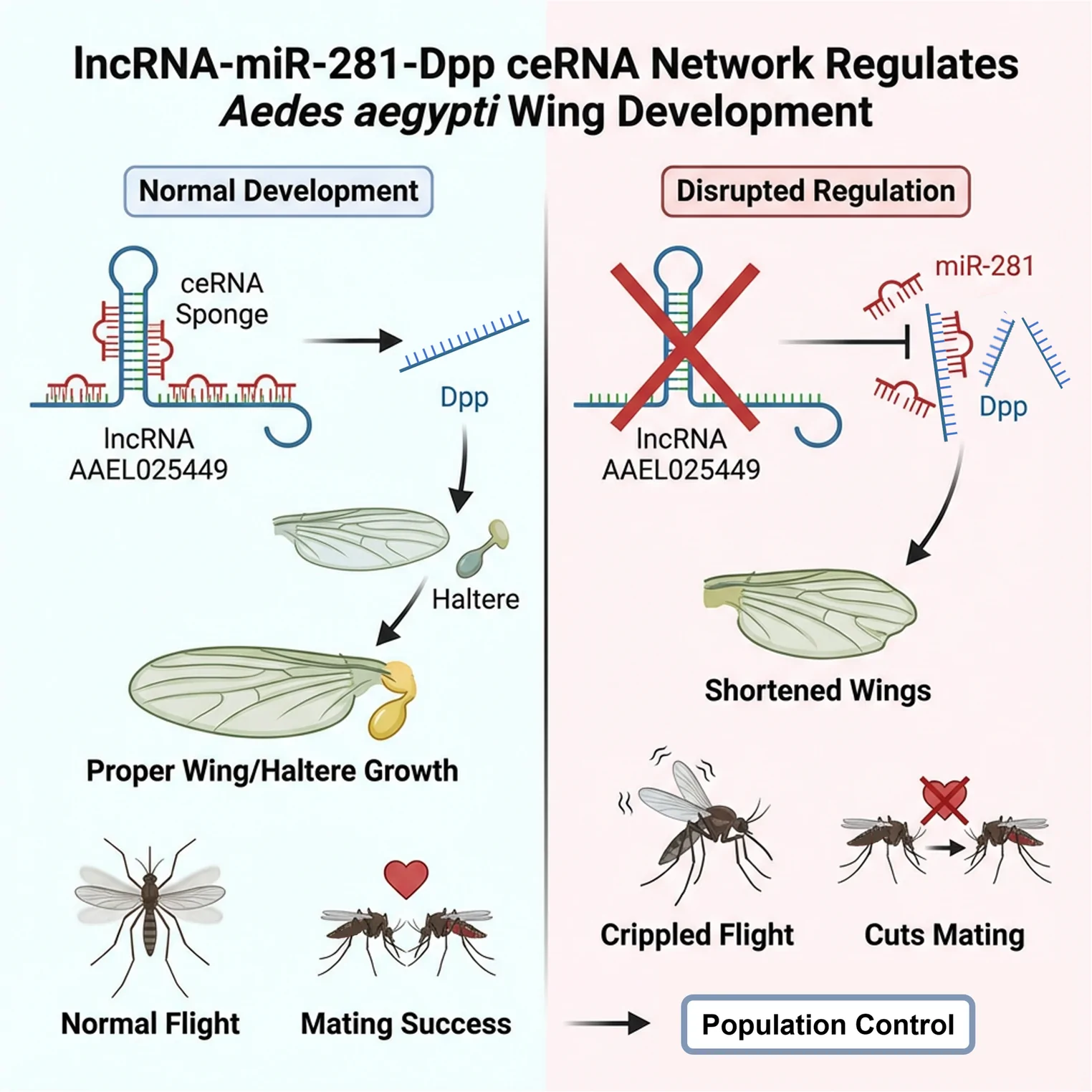
Schematic diagram of the mechanism

Ordered apoptosis of wing bud cells during the pupal stage is a fundamental process in insect wing development—critical for both normal morphogenesis and evolutionary adaptations such as wing degeneration [62, 63]. Precise temporal regulation of this process ensures proper wing formation, size, and shape, whereas dysregulation causes severe developmental defects [62–66]. Our study elucidates the spatiotemporal molecular mechanism by which the AAEL025449/miR-281/Dpp axis regulates *Ae. aegypti* wing bud development via the JNK pathway. Specifically, the wing bud/haltere-specific lncRNA AAEL025449 modulates Dpp levels during pupal wing development by competitively binding miR-281; Dpp then regulates JNK pathway activity via *brk*, maintaining normal wing bud cell apoptosis order. Both halteres and wings develop from wing buds, and these two organs are homologous [67, 68]. Given the highly similar expression patterns of AAEL025449/miR-281/Dpp in wings and halteres, coupled with our observation of direct evidence of key abnormal apoptosis during haltere development, the potential of AAEL025449/miR-281/Dpp to regulate haltere development is further underscored. These factors collectively affect *Ae. aegypti* flight capability, thereby indirectly compromising female host-seeking and male mating abilities (Fig. 7).

Spatially, AAEL025449 is a wing bud/haltere-specific lncRNA. Tissue-specific genes often confer novel developmental traits, making them promising targets for vector control [69–72]. miR-281, though non-wing bud/haltere-specific, specifically modulates Dpp expression in wing buds at 24 h PP, accurately transmitting AAEL025449’s regulatory signal. This may occur because other miRNAs competitively co-regulate Dpp in non-wing bud/haltere tissues [73, 74], mitigating miR-281’s effects on Dpp. Dpp is ubiquitously expressed in insect organs and tissues, closely linked to JNK pathway-mediated apoptosis activated by *brk* [21, 22, 48]. As a morphogen, Dpp regulates insect body size by driving and shaping organ growth patterns [75, 76]—consistent with the findings in Fig. 1 of this study. In *Drosophila*, Dpp’s requirements for diffusion and signal transduction vary across different wing regions and developmental stages [13, 77, 78]. Unlike Dpp regulation of wing development in *Drosophila* melanogaster, we observe that in *Ae. aegypti*, Dpp regulates the window of cell apoptosis in the Sc, R, and Cu veins around 24 h post-pupation (24 h PP), with apoptotic cells exhibiting a decreasing gradient from the anterior to posterior wing bud margin. Premature apoptosis at the leading edge of the wing bud may impair surface support and reduce flight stability.

Temporally, studies on *Ae. aegypti* and related Diptera show that wing bud cell apoptosis (programmed cell death) is most active during the early-to-middle pupal stage (20–34 h PP) [7]. Consistent with existing work, the mechanism proposed here impacts apoptosis primarily at 24–36 h PP. During the first 12 h PP, AAEL025449/miR-281/Dpp and *brk* remained in relative balance, demonstrating no significant regulatory function throughout the non-apoptotic phase. During 12–24 h PP, *brk* expression gradually increases in wing buds, preparing for the activation of apoptosis in less viable cells. Due to delayed regulation in the cascade [79–81], c-Jun (a JNK substrate) remains at low levels during this phase, keeping wing bud cell apoptosis rates low. Meanwhile, AAEL025449 expression increases, sequestering more miR-281 and gradually relieving translational repression of Dpp. At 24–36 h PP, more less viable cells enter JNK-mediated apoptosis, with c-Jun expression rising. The delayed inhibitory effect of high Dpp levels on *brk* becomes apparent, balancing apoptosis rates and preparing for the end of the apoptotic phase. From this stage until pre-eclosion, AAEL025449/Dpp/*brk*/c-Jun all show gradual downregulation. AAEL025449/miR-281/Dpp no longer significantly affects *brk*/c-Jun expression or apoptosis—indicating the proposed mechanism is only active during the early-to-middle pupal stage and becomes ineffective as wing development matures. This aligns with findings from other researchers highlighted earlier in this Sect [7]. In terms of upstream-downstream regulation, the approximately 12 h delayed Dpp/*brk*/c-Jun regulation observed here aligns with existing studies on Dpp’s role in insect cell apoptosis via the JNK pathway [55, 57, 82].

The mechanism proposed here shows no significant sexual dimorphism, revealing novel phenotypes in both male and female individuals. Our study found that when experimental manipulations reduced the wing-to-body length ratio of mosquitoes, *Ae. aegypti* flight capability was significantly impaired, exerting significant impacts on reproduction—manifested as reduced host-seeking ability in females and reduced mating ability in males. Flight is fundamental to *Ae. aegypti* mating, as both sexes must fly to locate, court, and mate [83, 84]. Experimental reduction of male flight activity reduces mating rates, insemination success, and population size via a mate-finding Allee effect [85, 86]. When experimental manipulations proportionally reduced wing length with body size, flight-based reproductive behaviors were unaffected; however, the probability of successful male mating declined due to an exacerbated body size disparity between males and females. Smaller males prefer to mate with smaller females, and their mating success declines when mating with larger females [87, 88]. Reproductive success is a primary evolutionary driver in insects. Reduced mating rates from flight impairments thus provide a strong molecular framework for understanding the adaptive evolution of wing plasticity.

While our study preliminarily elucidates the molecular mechanism by which AAEL025449/miR-281/Dpp regulates wing development in *Ae. aegypti*, it has limitations. Given the broad regulatory impact of Dpp, we cannot fully exclude the possibility that this axis influences physiological states via other tissues, despite the negligible expression of AAEL025449 therein. Due to the complex structure of early pupal wing bud development, we struggled to isolate intact wing bud tissues at 12 h PP and halteres at 12/24 h PP to fully characterize apoptosis staining across all stages. Possibly due to technical limitations, we have not yet observed significant differential phenotypes exhibited by halteres in adults. c-Jun mRNA levels were assessed as one of the observed outcomes in this study; however, its phosphorylation remains the canonical indicator of JNK pathway activation. In flight induction assay videos, many mosquitoes were not flying. This may stem from two factors. First, intragroup variability in mosquito blood-feeding response timing drove differential responses to the synthetic human odor source. Second, the synthetic human odor source had limited efficacy and required optimization. Although significant differences were observed between groups in our flight assays, these findings represent relative differences only, given the inherent discrepancies between our laboratory setting and natural field conditions. The current study remains at a theoretical stage, and its application for vector control requires further exploration. Nevertheless, our findings refine the interpretation of the wing bud/haltere-specific molecular regulatory mechanism of the classical Dpp pathway and offer additional insights into mosquito-borne disease control.

## Supplementary Information

Below is the link to the electronic supplementary material.

Supplementary Material 1

Supplementary Material 2

Supplementary Material 3

Supplementary Material 4

Supplementary Material 5

## Data Availability

The original data are included in the article. For additional information, inquiries can be directed to the corresponding authors.
