# Supplementary material for: LncRNA modulates Dpp-mediated wing development to influence flight in Aedes aegypti
Source: Cell Mol Life Sci. 2026 Jun 30;83(1):333. doi: 10.1007/s00018-026-06325-8 (PMC13582776; doi:10.1007/s00018-026-06325-8)
Supplement: Supplementary file 1 [file 18_2026_6325_MOESM5_ESM.docx]

**Supplementary information (SI): Figures S1-S12; Videos S1-S4; Tables S1, S2**

**Figure S1. Interference with Dpp induces physiological changes in the whole organism. A** qPCR analysis of Dpp expression trends in the wing buds of iLuc and iDpp models; significance analyses were performed by comparing experimental groups with controls at the same time point. **B** Statistical analysis of wing-to-body length ratios across different groups. **C** Imaging of pupal body size in iLuc and iDpp mosquitoes at 36 h PP (scale bar: 500 µm).

Data are represented as means ± SEM. ‘ns’ indicates *p* > 0.05; ‘*’ indicates *p* < 0.05; ‘**’ indicates *p* < 0.01; ‘***’ indicates *p* < 0.001.

**Figure S2. Expression analysis of non-coding RNAs in wing bud and non-wing bud tissues at 24 hours post-pupation (PP).**

Data are represented as means ± SEM.

**Figure S3. Computational prediction algorithms identified putative miR-281 binding sites in Dpp and AAEL025449.**

**Figure S4. qPCR analysis of AAEL025449 and miR-281 expression levels in the wing buds of iLuc and iDpp models at 24 h PP.**

Data are represented as means ± SEM. ‘**’ indicates p < 0.01; ‘***’ indicates p < 0.001.

**Figure S5. Schematic diagrams of the dual-luciferase reporter gene plasmids (RL: Renilla** **Luciferase; FL: Firefly Luciferase).**

**Figure S6. Validation of the knockout (KO) strain. A** Sequence alignment of the sgRNA-targeted genomic locus. **B** qPCR validation of AAEL025449 knockout efficiency.

Data are represented as means ± SEM. ‘***’ represents ‘*p* < 0.001’.

**Figure S7. Phenotypic difference analysis.** **A** Comparison of wing size between wild-type (WT) and KO (scale bar: 500 µm). **B** Statistical analysis of wing lengths across different groups.

**Figure S8. qPCR analysis of miR-281 and Dpp expression levels in the wing buds of WT and KO at 24 h PP.**

Data are represented as means ± SEM. ‘**’ represents ‘*p* < 0.01’, ‘***’ represents ‘*p* < 0.001’.

**Figure S9. Wing apoptosis was assessed using TUNEL-based apoptosis detection (scale bar: 100 µm; blue fluorescence: DAPI; green fluorescence: TUNEL).**

Data are represented as means ± SEM. ‘ns’ indicates *p* > 0.05.

**Figure S10. qPCR quantification of core apoptotic regulator expression profiles in wing buds at different time points.**

Data are represented as means ± SEM.

**Figure S11. Validation of the miR-281 overexpression model.** **A** qPCR analysis of miR-281 expression trends in the wing buds of mimic-NC and mimic-281 models; significance analyses were performed by comparing experimental groups with controls at the same time point. **B** qPCR analysis of Dpp and AAEL025449 expression levels in the wing buds of mimic-NC and mimic-281 models at 24 h PP. **C** Statistical analysis of wing lengths across different groups.

Data are represented as means ± SEM. ‘**’ represents ‘*p* < 0.01’, ‘***’ represents ‘*p* < 0.001’.

**Figure S12. qPCR analysis of Dpp expression in non-wing bud/haltere tissues at 24 h PP in WT and KO, as well as mimic-NC and mimic-281 models.**

Data are represented as means ± SEM. ‘ns’ indicates *p* > 0.05.

**Figure S13. Validation of potential competing miRNAs targeting Dpp. A** qPCR analysis of miRNA expression in whole-body extracts of mimic-NC and mimic-281 models at 24 h PP. **B** qPCR analysis of miRNA expression levels in wing versus non-wing tissues at 24 h PP.

Data are represented as means ± SEM. ‘**’ represents ‘*p* < 0.01’, ‘*’ represents ‘*p* < 0.05’. ‘ND’ indicates Not Detected

**VideoS1.** Recording of induced flight in WT

**VideoS2.** Recording of induced flight in KO

**VideoS3.** Recording of induced flight in mimic-NC

**VideoS4.** Recording of induced flight in mimic-281

**Table S1** Sequences for dsRNA, sgRNA, qPCR and PCR primers.

| *Primer name* | *Sequence (5'-3')* |
| --- | --- |
| RPS7 | F-TCAGTGTACAAGAAGCTGACCGGA |
|  | R-TTCCGCGCGCGCTCACTTATTAGATT |
| c-Jun | F-GTGCTGCATGGCTAATGCTC |
|  | R-ATGGAAGCGTTTACTCGCCA |
| *brk* | F-CACACCACAGTTCAAGCTGC |
|  | R-TCTGGTGGTGAATGTGTCGG |
| AAEL025449 | F-GTGCTGCCTCAGACCAGATT |
|  | R-TTCGGGCAAAACCAGGACTT |
| IAP5 | F-GACACCTCCGTCTGTTTCGT |
|  | R-CCTTCAGGGCGGTATAGCTG |
| IAP2 | F-CAGCAGGGCCTAACTTGTCA |
|  | R-ACAGCTGAAGATGTGGAGGC |
| IAP6 | F-AGTACCTCCAGGCGAGGAAT |
|  | R-GGTTGTTGGTCGAGGCAGTA |
| IAP1 | F-GAAGATGAACCGTGGGAGCA |
|  | R-ACACTCAAGCTCGACGACTG |
| Dronc | F-CCTCAAAGCCACCGGATTCT |
|  | R-CAGCTTTGGGCAATTGGCAT |
| Ark | F-CTCCTCCTGTCTTCAGCAGC |
|  | R-CCGAACCCGCCATATACGAA |
| Dpp | F-CGGGCTCCATCCTTTCTGTT |
|  | R-AGAGTCCGTGGTTCTGCTTG |
| U6 | ACATCGGTGGTGTTGATT |
| miR-281 | TGTCATGGAATTGCTCTCTTTA |
| miR-92b | AGGTCGTGACTTGTGCCCGTTTG |
| miR-263a | AATGGCACTGGAAGAATTCACGG |
| miR-11895 | TTCATGCATTCACTTCTGCTGA |
| miR-989 | TGTGATGTGACGTAGTGGTAC |
| AAEL025449-CRISPR-F | GAAATTAATACGACTCACTATAGGTACTCTAAAGAGAGTAACCAGTTTTAGAGCTAGAAATAGC |
| CRISPR Universal reverse primer | AAAAGCACCGACTCGGTGCCACTTTTTCAAGTTGATAACGGACTAGCCTTATTTTAACTTGCTATTTCTAGCTCTAAAAC |
| Dpp(wt)-CHECK2 | F-TAGGCGATCGCTCGAATTGTAAACTTTTACTCGTAG  CCAGGAGGGCGATGTAGTGCGCA |
|  | R-AATTCCCGGGCTCGAGTTTACATTATAAAACAACCA  TCTTGCGCACTACATCGCCCTCC |
| Dpp(mut)-CHECK2 | F-TAGGCGATCGCTCGAATTGTAAACTTTTACTCGTAG  CCAGGAGAATGGTGTAGAACGGG |
|  | R-AATTCCCGGGCTCGAGTTTACATTATAAAACAACCA  TCTCCCGTTCTACACCATTCTCC |
| AAEL025449(wt)-CHECK2 | F-TAGGCGATCGCTCGATTTGACATTTGACAGTGTTAC  TCTAAAGAGAGTAACCATGGC |
|  | R-AATTCCCGGGCTCGATGGAACTGCTCAAAAGTGAG  TAAAGCCATGGTTACTCTCTTTAG |
| AAEL025449(mut)-CHECK2 | F-TAGGCGATCGCTCGATTTTGACATTTGACAGTGTTA  CTCTAAAGAGAGTAATTGCTAT |
|  | R-AATTCCCGGGCTCGATGGAACTGCTCAAAAGTGAG  TAAAATAGCAATTACTCTCTTTAG |
| RNAi-Dpp | F-TAATACGACTCACTATAGGGAGAGCAAGAGCAAGC  GAAAACCA |
|  | R-TAATACGACTCACTATAGGGAGAACGATCCAATCG  CTCCATCC |

**Table S2** Key resources table

| REAGENT or RESOURCE | SOURCE | IDENTIFIER |
| --- | --- | --- |
| **Biological samples** | | |
| *Aedes aegypti* | Lab stock | N/A |
| White-feathered broilers | Lab stock | N/A |
| S2 cells | Lab stock | N/A |
| **Chemicals and peptides** | | |
| Cas9 protein | PNA Bio | CP01 |
| miRNA mimic/inhibitor | Sango | N/A |
| psiCHECK-2 Vector | Promega | C8021 |
| T4 DNA Ligase | NEB | M0202V |
| Trizol | Vazyme | R411 |
| Nile red | Sango | A606340 |
| **Critical commercial assays** | | |
| MEGAscript™ T7 Transcription Kit | Invitrogen | AM1334 |
| MEGAclear™ Transcription Clean-Up Kit | Invitrogen | AM1908 |
| FastPure Tissue/Bacteria DNA Isolation Mini Kit | Vazyme | DC112 |
| Taq Pro Universal SYBR qPCR Master Mix | Vazyme | Q712 |
| HiScript III RT SuperMix for qPCR (+gDNA wiper) | Vazyme | R323 |
| miRNA First-Strand cDNA Synthesis SuperMix | Transgen | AT351 |
| Lipofectamine 3000 | Invitrogen | L3000015 |
| APO™ One-Step TUNEL Apoptosis Kit | Ribo | C11012 |
| Dual-Luciferase® Reporter Assay System | Promega | E1910 |
| **Software and algorithms** | | |
| Prism 8 | GraphPad | https://www.graphpad.com |
| ZEN 3.1 | Carl Zeiss | https://www.zeiss.com/microscopy/en/products/software/zeiss-zen.html |
